# Regulation of Wnt Signaling Pathways at the Plasma Membrane and Their Misregulation in Cancer

**DOI:** 10.3389/fcell.2021.631623

**Published:** 2021-01-21

**Authors:** Yagmur Azbazdar, Mustafa Karabicici, Esra Erdal, Gunes Ozhan

**Affiliations:** ^1^Izmir Biomedicine and Genome Center, Dokuz Eylul University Health Campus, İzmir, Turkey; ^2^Izmir International Biomedicine and Genome Institute (IBG-Izmir), Dokuz Eylul University, İzmir, Turkey; ^3^Department of Medical Biology and Genetics, Faculty of Medicine, Dokuz Eylul University, İzmir, Turkey

**Keywords:** Wnt, frizzled, plasma membrane, cancer, lipid raft

## Abstract

Wnt signaling is one of the key signaling pathways that govern numerous physiological activities such as growth, differentiation and migration during development and homeostasis. As pathway misregulation has been extensively linked to pathological processes including malignant tumors, a thorough understanding of pathway regulation is essential for development of effective therapeutic approaches. A prominent feature of cancer cells is that they significantly differ from healthy cells with respect to their plasma membrane composition and lipid organization. Here, we review the key role of membrane composition and lipid order in activation of Wnt signaling pathway by tightly regulating formation and interactions of the Wnt-receptor complex. We also discuss in detail how plasma membrane components, in particular the ligands, (co)receptors and extracellular or membrane-bound modulators, of Wnt pathways are affected in lung, colorectal, liver and breast cancers that have been associated with abnormal activation of Wnt signaling. Wnt-receptor complex components and their modulators are frequently misexpressed in these cancers and this appears to correlate with metastasis and cancer progression. Thus, composition and organization of the plasma membrane can be exploited to develop new anticancer drugs that are targeted in a highly specific manner to the Wnt-receptor complex, rendering a more effective therapeutic outcome possible.

## Introduction

The Wnt signaling pathway is an evolutionarily conserved signal transduction cascade that controls a wide range of biological events from embryonic development to tissue regeneration (Nusse, 2005; Aman et al., 2018; Steinhart and Angers, 2018). The pathway is broadly divided into two branches as the canonical (β-catenin-dependent) Wnt signaling and the non-canonical (β-catenin-independent) Wnt signaling, which is further branched into the Wnt/planar cell polarity (PCP) and the Wnt/Ca^2+^ pathways. Wnt signaling pathways play a multitude of essential roles in cell fate determination, cell polarity, cell migration and patterning during embryonic development, adult tissue homeostasis and regeneration of various tissues and organs. Consequently, misregulation of Wnt signaling has been associated with a variety of human diseases including developmental defects, degenerative diseases, and many cancers. Although numerous components of the Wnt pathways have been characterized for their functional roles, many questions related to tight regulation and modifications of signaling remain unanswered. Besides, despite many efforts, so far no drugs have been approved to specifically target the pathway, leading to a gap in targeted therapy of Wnt-related diseases (Nusse and Clevers, 2017; Krishnamurthy and Kurzrock, 2018). A better understanding of the molecular mechanisms underlying Wnt-mediated cellular responses is especially critical for development of effective anticancer drugs that are expected to interfere with the highly activated Wnt signaling in many cancers (Jung and Park, 2020).

**FIGURE 1:**
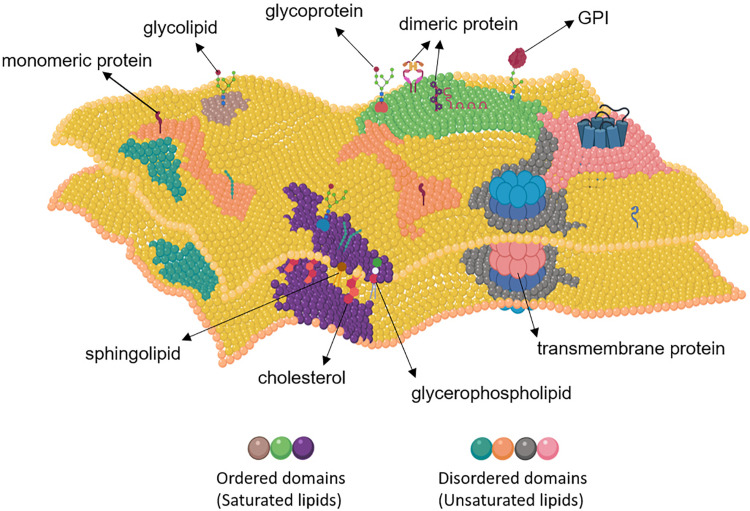
Structure of the plasma membrane. The plasma membrane consists of ordered and disordered domains. Basically, ordered domains are enriched in cholesterol, sphingolipids, glycerophosholipids, glycosylphosphatidylinositol (GPI)-anchored proteins, glycoproteins, dimeric proteins, and saturated lipids, whereas the disordered domains usually contain monomeric and transmembrane proteins and unsaturated lipids. Created with BioRender.com.

The plasma membrane plays a dual central role in regulation of cell signaling. On the one hand, it acts as a barrier and ensures spatial segregation between extracellular environment and the cytosolic compartment. At the same time, by harboring many cell surface receptors and regulators that are involved in cell signaling, it actively controls transmission of molecular signals from the exterior to the interior of a cell and precisely links them to downstream signaling events. The plasma membrane is likewise vital for initiation of Wnt signaling where Wnt ligands bind to their receptor complexes in specialized membrane domains that are considered as dynamic assemblies of various saturated lipids, sterols and lipid-anchored proteins (Sezgin et al., 2017a, b, 2015) (Figure 1). Owing to their central roles in initiation of signaling, Wnt pathway components acting at the plasma membrane have been frequently investigated as drug targets (Gurney et al., 2012; Krishnamurthy and Kurzrock, 2018; Zeng et al., 2018a). Since plasma membranes of healthy and diseased cells display major structural differences, the influence of membrane nanoenvironment on signaling function should be considered carefully when developing novel therapeutic approaches that target Wnt pathways at the membrane.

Here, we review the cellular mechanisms underlying regulation of Wnt signaling pathways at the plasma membrane domains with a specific focus on the role of lipid molecules. Next, we address how Wnt pathways are misregulated at the plasma membrane through their ligands, receptors and membrane associated pathway modulators in cancer. Since Wnt signaling has been associated with numerous cancers, here we will focus on four common cancers, i.e., lung, colorectal, liver, and breast cancers. We also discuss the therapeutic approaches that aim to inhibit aberrant signaling activity in cancer by targeting the Wnt-receptor complex at the plasma membrane.

## Wnt Signaling Pathways

Wnt signaling pathway regulates proliferation, survival, polarity, and migration, differentiation of cells as well as maintenance of stem cells of various lineages during embryonic development and tissue homeostasis (Clevers et al., 2014). Examples come from the stem cells that reside in different regions of the body including the digestive tract, hematopoietic system and the nervous system (Lee et al., 2000; Willert et al., 2003; Nusse et al., 2008). The Wnt target gene Lgr5 has been identified as a stem cell marker at the crypts of small intestine and colon (Barker et al., 2007). Further studies have revealed that Lgr5 and Axin2 are expressed in the stem cells of multiple organs such as the liver, mammary gland, stomach, brain, kidney, cochlea and ovary and associated with widespread self-renewal capacity (Barker et al., 2010, 2012; van Amerongen et al., 2012; Bowman et al., 2013; Flesken-Nikitin et al., 2013; Huch et al., 2013; Jan et al., 2013). Thus, by ensuring the formation of the stem cell pool and its continuity via asymmetric divisions, Wnt signals play critical roles in establishing niches for stem cells in various tissues and organs and determining molecular programs of tissue regeneration.

The Wnts consist of a large family of protein ligands that interact with a number of receptors and co-receptors at the plasma membrane. Based on 19 Wnt ligands and 10 Fz receptors in mammals, these interactions constitute one of the most complex relationships between extracellular ligands and cell surface receptors (Clevers and Nusse, 2012). While some Wnt ligands are associated with a particular Wnt signaling pathway, others that cannot easily be attributed to a single Wnt pathway are competent to initiate signaling in more than one branch.

### Wnt/β-Catenin Signaling: The Canonical Wnt Pathway

**FIGURE 2:**
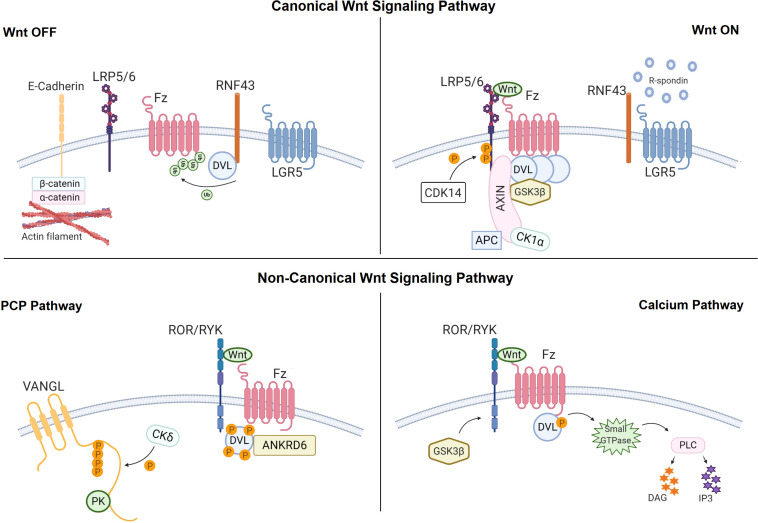
The Wnt signaling pathways at the plasma membrane. Canonical Wnt signaling pathway: In the Wnt OFF state, defined by the absence of canonical Wnt ligand, β-catenin is phosphorylated by the destruction complex that is comprised of glycogen synthase kinase 3β (GSK3β) and casein kinase 1α (CK1α), Axin and dishevelled (DVL) and Adenomatous Polyposis Coli (APC). Ring finger protein 43 (RNF43) also inhibits the pathway by binding the Fz receptor via DVL. In the Wnt ON state, canonical Wnt ligand binds to the Frizzled (Fz) receptor and the low-density lipoprotein receptor-related protein 5/6 (LRP5/6) co-receptor. This interaction recruits DVL and AXIN to the Wnt-receptor complex and leads to disassembly of the destruction complex, causing stabilization of β-catenin in the cytosol. Binding of R-spondin to leucine-rich repeat-containing G-protein coupled receptor 5 (LGR5) enhances Wnt signaling activity by neutralizing RNF43. Non-canonical Wnt signaling pathways: In the PCP pathway, non-canonical Wnt ligands bind to the ROR/RYK-Fz receptor complex and recruit DVL. Next phosphorylation of VANGL activates the pathway. In calcium pathway, binding of non-canonical Wnt ligands to ROR/RYK-Fz recruits DVL and activated DVL binds to the small GTPase that further activates phospholipase C (PLC). Created with BioRender.com.

Being the most studied Wnt pathway, canonical Wnt signaling operates through cytoplasmic accumulation of β-catenin protein and is essential for embryonic development, adult homeostasis and stem cell maintenance (Nusse and Clevers, 2017). In the absence of canonical Wnt ligand, β-catenin is phosphorylated by a multiprotein destruction complex that includes the serine-threonine kinases glycogen synthase kinase 3β (Gsk3β) and casein kinase 1α (Ck1α), the scaffold protein Axin and the cytoplasmic effector proteins dishevelled (Dvl) and Adenomatous Polyposis Coli (APC), and targeted for degradation by the ubiquitin-proteasome system (Kimelman and Xu, 2006; MacDonald et al., 2009). Active canonical Wnt ligand, however, binds to its cell surface receptor frizzled (Fz) and co-receptor low-density lipoprotein receptor-related protein 5/6 (Lrp5/6) and recruits Dvl and Axin to the plasma membrane, leading to disassembly of the destruction complex (Figure 2). β-catenin then accumulates in the cytoplasm, translocates into the nucleus and interacts with the T-cell factor/lymphoid enhancer factor (TCF/LEF) family of transcription factors to regulate the expression of target genes (Logan and Nusse, 2004; Taketo, 2004; Nusse, 2005; Angers and Moon, 2009; Clevers and Nusse, 2012).

### The Non-canonical Wnt Pathways

Acting independently of β-catenin, the non-canonical Wnt/PCP pathway coordinates cell movement and tissue polarity via small GTPase RhoA or c-Jun N-terminal kinase (JNK) (Katoh, 2005). The typical non-canonical ligands Wnt5a, Wnt5b, and Wnt11 initiate the PCP signaling via interacting with their receptors Fz3 or Fz6 and co-receptors Ror1, Ror2, or Ptk7 (Humphries and Mlodzik, 2018). The human planar cell polarity proteins Vangl1, Vangl2, Prickle1, and Prickle2, Cadherin EGF LAG seven-pass G-type receptors (Celsr1, Celsr2, Celsr3), Dvl1, Dvl2, Dvl3, and Ankyrin repeat domain 6 (Ankrd6) are involved in the signaling cascade (Figure 2). Dvl-dependent Wnt/PCP signals are transduced through the Dishevelled-associated activator of morphogenesis (Daam1 and Daam2) proteins or Mitogen activated protein (MAP) kinase kinase kinase (MAPKKK) and MAPKK4/7 and activate the Ras homolog family member A-Rho-associated protein kinase (RhoA-Rock) or Rac-JNK signaling cascades, respectively. On the other hand, the Dvl-independent G protein-dependent non-canonical Wnt pathway operates through G proteins, Receptor tyrosine kinases (RTKs) and Phospholipase C (PLC), leading to intracellular calcium release, activation of the serine/threonine protein phosphatase calcineurin and accumulation of Nuclear factor of activated T cells (NFAT) in the nucleus (Katoh, 2005; Kohn and Moon, 2005; Katoh and Katoh, 2017). Elevated cytoplasmic calcium can also activate Nemo-like kinase (NLK) signaling pathway through Calcium/calmodulin dependent kinase II (CaMKII).

### Regulation of Wnt Pathways at the Plasma Membrane

Both canonical and non-canonical Wnt signaling pathways are tightly regulated by a number of modulators that are evolutionarily conserved and function either extracellularly to regulate ligand-receptor interactions or intracellularly to modify cytosolic or nuclear components of the pathway. Extracellular modulators can be either secreted, membrane-bound or transmembrane proteins and are broadly classified into two groups according to their functions: (1) The agonists that include Norrin, R-spondins (Rspos), GPI-anchored membrane proteins Ly6/Plaur domain-containing 6 (Lypd6) and Reck, G protein-coupled receptors Gpr124 and Gpr37 and (2) the antagonists that include secreted Dickkopf proteins (Dkks), Secreted frizzled-related proteins (Sfrps), secreted Wnt-inhibitory factor 1 (Wif-1), secreted Wise/Sost proteins, secreted protein Cerberus, secreted insulin-like growth-factor binding protein 4 (Igfbp-4), secreted palmitoleoyl-protein carboxylesterase Notum, single-transmembrane proteins Shisa, Wnt-activated inhibitory factor 1 (Waif1/5T4), adenomatosis polyposis coli down-regulated 1 (Apcdd1), membrane-bound metalloprotease Tiki1, transmembrane E3 ubiquitin ligase Zinc and ring finger 3 (Znrf3) and its functional homolog Ring finger protein 43 (Rnf43), (Cruciat and Niehrs, 2013; Jiang and Cong, 2016; Berger et al., 2017; Eubelen et al., 2018). Owing to their critical roles in ligand or receptor modification, ligand-receptor complex formation, regulation of signaling activity in membrane subdomains and receptor internalization, these secreted and membrane proteins are noteworthy candidates for therapeutic targeting of Wnt signaling pathways.

## Initiation of Wnt Signaling at the Plasma Membrane

The plasma membrane has a complex heterogeneous and highly dynamic structure that is composed of lipids and proteins with varying features and compartmentalized into numerous smaller structures called micro- or nanodomains (Eggeling et al., 2009; Owen et al., 2012; Sarmento et al., 2020). This heterogeneity leads to formation of ordered membrane domains, also known as lipid rafts or lipid nanodomains, which are assembled from saturated lipids, sphingolipids, sterols, glycolipids, glycoproteins and certain lipid-anchored proteins such as glycosylphosphatidylinositol (GPI)-anchored proteins and fatty acylated proteins, leaving the relatively disordered domains occupied by unsaturated lipids and a large fraction of membrane proteins (Sezgin et al., 2015, 2017b; Fakhree et al., 2019; Kusumi et al., 2020; Figure 1). The ordered domains of the plasma membrane have been shown to be essential for cell signaling events by controlling membrane-cytoskeleton communication, ligand-receptor interaction and receptor clustering (Simons and Toomre, 2000; Thomas et al., 2004; Ozhan et al., 2013; Agarwal et al., 2018; Azbazdar et al., 2019). For example, activation of epidermal growth factor receptor (EGFR) signaling by the epidermal growth factor (EGF) has been proposed to induce coalescence of different lipid rafts and formation of signaling platforms (Hofman et al., 2009; Irwin et al., 2011). After exposure to the Gram-negative bacterial cell wall component lipopolysaccharides, Toll-like receptor 4 (TLR4) of the immune system cells likewise interacts with its sorting adaptor Toll/interleukin-1 receptor domain-containing adaptor protein (TIRAP) in the lipid rafts (Barnett and Kagan, 2020).

### Activation of Canonical Wnt Signaling in the Plasma Membrane Domains

Interaction of the canonical Wnt ligand with its (co)receptors triggers Lrp6 phosphorylation and endocytosis of receptor complexes, both of which appear to occur preferentially in the ordered membrane domains (Yamamoto et al., 2008; Sakane et al., 2010). While appearing in a way unexpected due to the fact that majority of the receptor Fz and the co-receptor Lrp6 are preferentially localized in the disordered membrane domains, both processes are facilitated by the GPI-anchored Lypd6 protein that interacts with Lrp6, helps it to associate with the ordered membrane domains via its GPI anchor and enhances Wnt/β-catenin signaling by promoting Lrp6 phosphorylation in these domains (Ozhan et al., 2013). Lipid raft disruption via induction of cholesterol efflux from the membrane with methyl-β-cyclodextrin significantly reduces Lrp6 phosphorylation in neuroblastoma cells, indicating that membrane order and integrity is essential for Lrp6 activity (Riitano et al., 2020). Further work has revealed that the canonical Wnts bind to their receptor complexes selectively in the ordered membrane domains (Sezgin et al., 2017a). Disruption of the membrane order *in vitro* or *in vivo* using inhibitors that specifically target lipids associated with the ordered domains significantly reduces canonical Wnt signaling activity, underscoring the influence of the plasma membrane lipid content on early interaction of the canonical Wnt with its receptors and downstream signaling activity (Sezgin et al., 2017a). Presence of canonical Wnt ligand stimulates enrichment of cholesterol, an essential component of the ordered membrane domains, in the inner membrane leaflet and receptor clustering that is likely aided by other membrane components such as heparin sulfate and phosphatidylinositol-4,5-bisphosphate (PIP2) (Mii et al., 2017; Erazo-Oliveras et al., 2018). Defects in synthesis of cholesterol thus reduces Wnt signaling and results in abnormalities in craniofacial development and neural crest cell differentiation (Sezgin et al., 2017a; Castro et al., 2020). The glycosphingolipid mannosyl glucosylceramide, which assembles with sterols into ordered membrane domains, likewise enhances presynaptic Wnt1/Wingless (Wg) signaling in the lipid rafts and promotes synaptic bouton formation at the *Drosophila* neuromuscular junction (Huang et al., 2018). Wnt/β-catenin signaling can also act in combination with Reactive Oxygen Species (ROS) signaling to regulate nuclear β-catenin levels during neural differentiation in a lipid raft dependent manner (Haack et al., 2015).

Regulation of Wnt-receptor interaction in ordered domains through membrane proteins appears not to be restricted to canonical Wnt signaling. The heparan sulfate proteoglycan Glypican-4 (Gpc4) is one such bifunctional molecule that controls canonical and non-canonical Wnt pathways by binding to Wnt3a and Lrp6 in the ordered domains and to Wnt5a and Ror2 in the disordered domains, respectively (Sakane et al., 2012). Likewise, autocrine Wnt10b has been shown to be drafted to the ordered domains by the fibroblast-derived (FD) exosomes and activate mTOR signaling that in turn promotes axonal regeneration independently of β-catenin (Tassew et al., 2017). Conversely, inhibition of fatty acid synthase (FASN), the major source of long-chain fatty acids such as palmitate, interferes with lipid biosynthesis, disrupts ordered membrane architecture and inhibits Wnt/β-catenin signaling along with PI3K-AKT-mTOR pathways, most likely also inhibiting protein lipidation that is necessary for proper signal transduction (Ventura et al., 2015).

Apart from the components of the Wnt-receptor complex, *N*-terminally dephosphorylated (dephospho) β-catenin, the *de novo* synthesized form of β-catenin that correlates with Wnt signaling activity, has been reported to colocalize at the plasma membrane with Lrp6 and two members of the destruction complex, namely APC and Axin, shortly after Wnt stimulation and independently of E-cadherin (Hendriksen et al., 2008). Routing of dephospho-β-catenin to the membrane via the Wnt receptor complex appears to constitute a key step in regulation of its transcriptional activity and Wnt signaling. The activity of β-catenin as a membrane component may be controlled by other molecules under certain ambient conditions. For example, non-muscle myosin II induces accumulation of cortical F-actin and E-cadherin to the adherens junctions, resulting in corecruitment of β-catenin to the membrane to maintain cellular contraction and inhibition of Wnt signaling due to reduced levels of cytoplasmic β-catenin (Hall et al., 2019). Cytoskeletal networks have also been reported to regulate Wnt signaling activity through plasma membrane domains during differentiation of stem cells (von Erlach et al., 2018).

### Wnt Signalosome and the Role of Endocytosis

Interaction of Wnts ligands with their surface receptors within particular membrane domains activate an immediate biochemical response that triggers internalization of the Wnt signalosome, a dynamic signaling complex assembled by Dvl upon formation of the Wnt-receptor complex (Bienz, 2014; Gammons et al., 2016). Internalization of signalosome is essential for pathway activation that is balanced by degradation of excessive ligands and clearance of surface receptors, ultimately downregulating the signaling (Feng and Gao, 2015). At the same time, paradoxically, receptor-mediated endocytosis negatively regulates Wnt signaling through internalization and degradation of the receptor complex. The route of endocytosis is determined by multiple factors including the types of ligands and receptors, other molecules in the environment, feedback regulatory mechanisms and how the signal is terminated.

Canonical Wnt-mediated receptor complex is known to be endocytosed via the clathrin-independent route in the smooth invaginations of the plasma membrane, the so-called caveolae, which form as subset of the ordered membrane domains (Parton et al., 2006; Yamamoto et al., 2006; Bilic et al., 2007; Gong et al., 2008). Tyrosine-based motifs within the cytoplasmic tail of Lrp6 has been shown to be essential for determining its distribution within the membrane domains and its internalization routes, which is critical to keep signal activity under control (Liu et al., 2014). However, a large body of evidence supports that clathrin-dependent endocytosis of the Wnt-receptor complex can also enhance β-catenin-dependent signaling and that clathrin is a prerequisite for Wnt signalosome formation (Kim et al., 2013; Hagemann et al., 2014; Gammons et al., 2016; Brunt and Scholpp, 2018). Endocytosis has been proposed to regulate Wnt/β-Catenin signaling mainly via four alternative ways that include early endosomal acidification of the co-receptor Lrp6 for pathway activation, sequestration of GSK-3 to limit β-catenin proteolysis, clearance of ubiquitin ligases that target Wnt receptors for degradation and facilitation of signaling by stabilization of Dvl (Brunt and Scholpp, 2018).

β-catenin-independent Wnt pathway operates through clathrin-dependent endocytic route that mediates uptake of PCP components together with Syndecans, the transmembrane proteoglycans (Yamamoto et al., 2008; Ohkawara et al., 2011). Wnt/PCP signaling regulates cell adhesion and migration by regulating internalization of cadherins via protocadherins during vertebrate gastrulation (Brinkmann et al., 2016; Brunt and Scholpp, 2018). Recent data obtained from plasma membrane capacitance recordings have further supported that non-canonical Wnt5a is exclusively endocytosed via clathrin-coated vesicles (Bandmann et al., 2019).

Endocytosis of the Wnt-receptor complex can also be affected by different molecules and pathways. For example, in addition to regulating proteolytic degradation of β-catenin as part of the destruction complex, APC keeps Wnt receptor internalization and pathway activation under control by forming a complex with clathrin (Saito-Diaz et al., 2018). AP2-associated kinase 1 (Aak1), which is activated by Wnt/β-catenin signaling, likewise promotes clathrin-mediated endocytosis of Lrp6 and thus negatively regulates the pathway (Agajanian et al., 2019). The mammalian target of rapamycin complex 1 (mTORC1) signaling has also been shown to inhibit Wnt/β-catenin signaling by suppressing the expression of membrane Fz through enhancing its Dvl-dependent clathrin-mediated internalization (Zeng et al., 2018b). Further mechanistic studies on the interplay between Wnt signaling and other molecules will provide a deeper understanding of the tight control of pathway activity.

### Posttranslational Modifications in Wnt Proteins

Wnts are 350–400 amino acid-long cysteine-rich secreted glycoproteins that are highly conserved in metazoans. Mammals have 19 Wnt ligands that act through the canonical or non-canonical Wnt pathway and thus contributes to the pathway specificity and complexity (Clevers and Nusse, 2012).

The high number of conserved cysteine residues that Wnt proteins harbor suggest that the intra- and inter- molecular disulfide bonds are important for the proper folding, multimerization and function of Wnt proteins (Tang et al., 2012). Wnts undergo two main types of posttranslational modifications, i.e., *N*-glycosylation and lipidation/acylation at the endoplasmic reticulum (ER) and are subsequently transported to the plasma membrane via the cargo protein Wntless (Wls)/Evi, which is essential for Wnt secretion (Glaeser et al., 2018; Gradilla et al., 2018). Various mutations introduced at N-linked glycosylation sites of different Wnt proteins have shown that glycosylation is essential for proper folding and secretion of Wnt proteins but dispensable for their signaling activity (Mason et al., 1992; Komekado et al., 2007; Kurayoshi et al., 2007; Tang et al., 2012).

#### The Role of Palmitoylation in Wnt Function

Being a prominent mode of lipidation, acylation of Wnts takes place at conserved amino acid residues through palmitoylation and is mainly catalyzed by the protein Porcupine, a membrane-bound O-acyltransferase of the ER (Willert and Nusse, 2012; Gao and Hannoush, 2014; Nile and Hannoush, 2016). Initial mass spectrometry-based studies on mouse Wnt3a propounded two different sites for addition of palmitoyl moieties, which were a thioester-linked palmitic acid at a conserved cysteine and an oxyester-linked palmitoleic acid at a conserved serine (Willert et al., 2003; Takada et al., 2006). While the conserved serine has been confirmed as the consensus acylation site across all Wnts by high-resolution crystal structure analysis of *Xenopus* Wnt8, the conserved cysteine was found to be engaged in a disulfide bond, thus preventing it from serving as a lipidation site (Janda et al., 2012). Interestingly, canonical Wnts have been proposed to be acylated by palmitoleic acid, a monounsaturated fatty acid, which is assumed to exhibit a kinked conformation and thus fit into the cavity of the Porcupine (Takada et al., 2006; Nile et al., 2017; Lee et al., 2018, 2019). However, considering the fact that palmitoylation targets soluble proteins into ordered membrane domains, palmitoylation of Wnt by a monounsaturated fatty acid contradicts with its preferential binding in the ordered domains and ability to activate signaling therein (Zhai et al., 2004; Levental et al., 2010; Ozhan et al., 2013; Sezgin et al., 2017a). Computational structural analyses have indeed supported that canonical Wnt is likely modified by palmitic acid, a saturated fatty acid, by adopting a conformation compatible with the stereochemical features of Wnt modification (Azbazdar et al., 2019).

The role of lipidation in Wnt secretion and functionality has been investigated by mutagenesis of the conserved palmitoylation sites of various Wnt proteins (Kurayoshi et al., 2007; Franch-Marro et al., 2008; Tang et al., 2012; Luz et al., 2014; Hosseini et al., 2019; Speer et al., 2019). For example, palmitoylation mutants of mouse Wnt1 and Wnt3a were found to be secreted at varying levels while their signaling activities were significantly and consistently reduced (Takada et al., 2006; Doubravska et al., 2011; Galli and Burrus, 2011; Gao and Hannoush, 2014). Acylation mutant of Drosophila Wg was likewise found to be secreted normally but with markedly weaker signaling activity (Franch-Marro et al., 2008). Strikingly, several non-acylated mutants of Wnt1, Wnt3a, and Wnt8 from different species have been identified to vary dramatically in their rates of secretion, interactions with the receptor Fz and abilities to activate signaling *in vitro* or *in vivo* (Speer et al., 2019). Studies on the zebrafish canonical Wnt ligands have reported that Wnt palmitoylation is essential for activation of signaling but may be dispensable for secretion (Luz et al., 2014; Azbazdar et al., 2019). APT1-mediated depalmitoylation has been shown to be important for asymmetric localization of β-catenin and Wnt signaling activity during development (Stypulkowski et al., 2018). Interestingly, *Drosophila* WntD does not appear to undergo any glycolysis and acylation (Ching et al., 2008). Therefore, the impact of acylation on the secretion and function of different types of Wnts appears to vary significantly. We believe that individual and context-dependent characterization of Wnt ligands will provide critical insight into the how Wnt signaling activity is modified in different types of cancers and how therapeutic approaches could be specifically designed.

### The Impact: How Can We Benefit From What We Have Learned So Far?

Wnt signaling pathways control a plethora of cellular responses involved in development, homeostasis and disease. Thus, the molecular interactions underlying initiation of Wnt pathways at the plasma membrane have the potential to serve as attractive drug targets, especially for cancer where Wnt signaling is broadly misregulated. The disclosure of the functional role of plasma membrane organization in Wnt/β-catenin signaling will shed light on how the membrane background can be exploited for therapeutic approaches:

(1) The drugs can be packed in specific lipid-based drug delivery systems to directly target them to the relatively ordered domains of the membrane where Wnt-receptor complex formation occurs.
(2) The peptide drugs can be modified by introducing particular lipid moieties that help them target Wnt-receptor complex more precisely. Posttranslational lipid modifications, in particular palmitoylation, might be a good candidate to enhance domain-specific receptor targeting.
(3) A very convenient strategy would be lipid fingerprinting of cancer cells to characterize membrane lipid profiles of individual cell types. This would enable selectively inhibition of aberrant activated Wnt signaling pathway by direct modification of membrane lipid composition.

Overall, we believe that unraveling plasma membrane organization with respect to (mis)regulation of Wnt signaling in health and disease will help not only develop new strategies on targeted anticancer therapies and but also increase target specificity of existing drugs that interfere with the ligand-receptor complex at the plasma membrane.

## Misregulation of Wnt Signaling Pathways at the Plasma Membrane in Cancer

Dysregulation of plasma membrane domains with respect to its structural organization and dynamics, disruption of membrane protein and lipid homeostasis or mutations in genes encoding for membrane proteins can cause misregulation of cell signaling events and promote oncogenic signaling activities. For example, breast cancer cell lines with high levels of epidermal growth factor receptor (EGFR) have been found to be resistant for the tyrosine kinase inhibitors (TKIs) targeting EGFR because of EGFR accumulation in the lipid rafts at the membranes of these cells and pharmacological depletion of cholesterol from the rafts decreased this resistance (Irwin et al., 2011). The constitutively active mutant form of the non-receptor tyrosine kinase Src has likewise been reported to accumulate in the lipid rafts of small cell lung cancer (SCLC) cells and stimulate oncogenic phosphoinositide 3-kinase (PI3K) signaling by facilitating the interaction of particular PI3K isoforms with Src kinases (Arcaro et al., 2007). Epithelial-to-mesenchymal transition (EMT) has also been associated with modulation of lipid raft properties strongly suggesting that alterations of membrane biophysical phenotypes are required to maintain metastatic potential of cancer cells (Tisza et al., 2016). In this section we will review how misregulation of Wnt signaling mainly in four common cancers; i.e., lung, colorectal, liver, and breast, is linked to the defective signaling at the plasma membrane and discuss the potential therapeutic approaches based on targeting the Wnt pathway at the membrane in these cancers.

### Lung Cancer

Being the most common cause of cancer-related death in the world, lung cancers are histologically classified as non-small-cell lung cancer (NSCLC), which comprises about 85% of lung cancers, and small-cell lung cancer (SCLC). The main subtypes of NSCLCs are adenocarcinoma (ADC), squamous cell carcinoma (SCC), and large cell carcinoma (LCC). Wnt signaling is frequently abnormally activated in lung cancers. Overexpression of Wnts1-3, Wnt5a, Wnt11, and Fz8 is common in NSCLC (Nakashima et al., 2012; Stewart, 2014; Huang et al., 2015; Rapp et al., 2016). Resected NSCLC samples with high levels of Wnt3 are characterized with a significantly higher Ki67 proliferation index and a significantly lower apoptotic index, resulting in a considerably lower survival rate in patients with high-Wnt tumors than in those with low-Wnt tumors (Nakashima et al., 2012). Wnt3a treatment has been found to decrease the expression of E-cadherin and increase that of N-cadherin and Vimentin, thereby promoting EMT and metastasis in NSCLC cells (Li et al., 2015). Intriguingly, overexpression of the cell surface heparan sulfate proteoglycan Glypican-5, which competitively binds to Wnt3a, inactivates Wnt/β-catenin pathway and consequently suppresses EMT and metastasis in lung ADC (Wang et al., 2016). Wnt7a and its receptor Fz9 are significantly downregulated in NSCLC compared to normal uninvolved lung tissue and, upon interaction; they likewise trigger a tumor suppressor pathway by inhibiting transformed cell growth and promoting epithelial differentiation through activation of JNK pathway but not the Wnt/ß-catenin pathway (Winn et al., 2005). On the other hand, upregulation of Ror 1, a member of the Ror family of RTKs, has been shown to promote lung carcinogenesis through activation of Wnt/PCP and Wnt/RTK signaling cascades (Katoh and Katoh, 2017).

#### Abnormalities in Wnt Ligands, Receptors and Pathway Modulators in Lung Cancer

**FIGURE 3:**
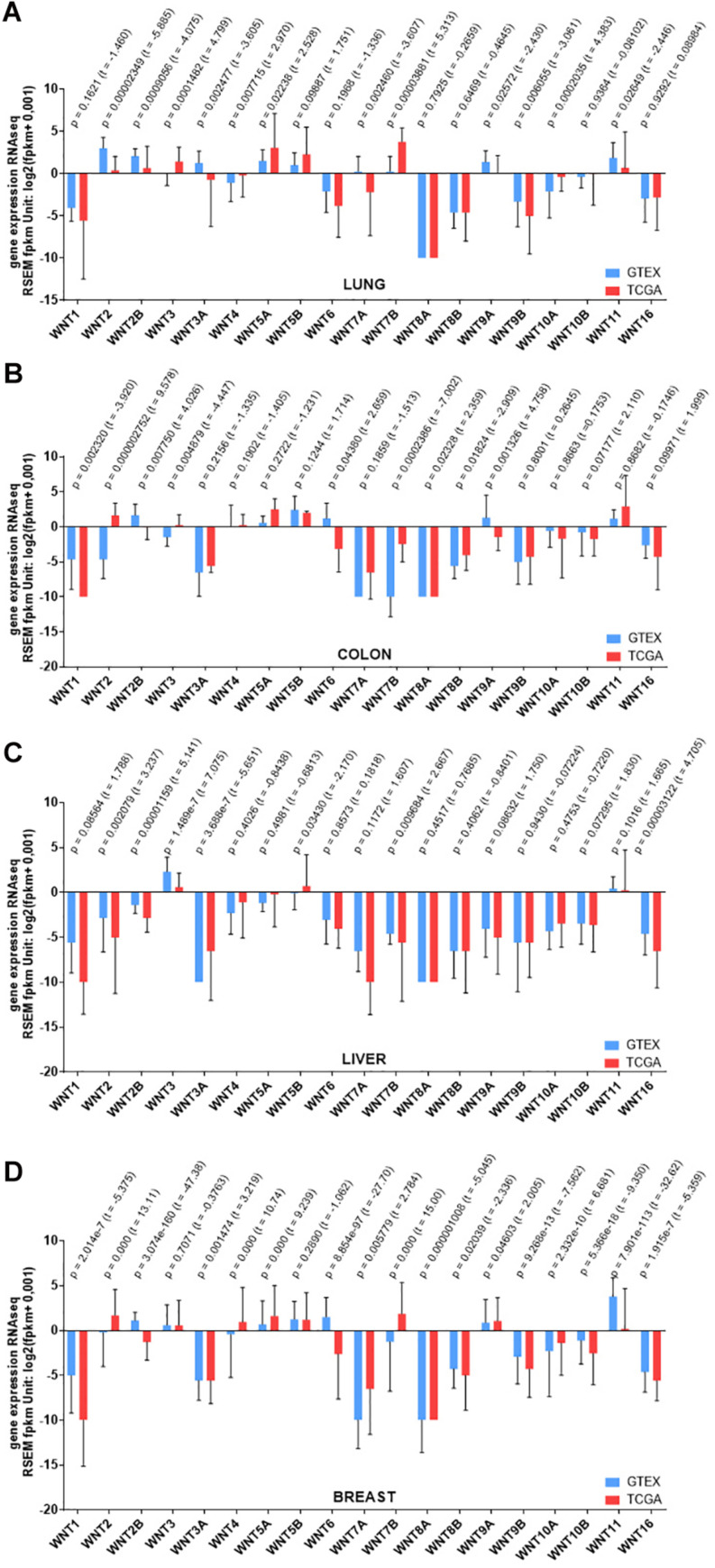
RNAseq gene expression levels of Wnt ligands in different cancers. **(A)** Lung cancer **(B)** Colon cancer **(C)** Liver cancer **(D)** Breast cancer. The data were downloaded from https://xenabrowser.net/on August 10, 2020. For each cancer type, data were filtered from the browser extension data set on the TCGA TARGET GTEx dataset, which includes approximately 13 thousand genes. Studies were obtained from GTEx project and TCGA. Gene expressions were analyzed using RSEM fpkm method. Significance was calculated using Welch’s *t*-test and graphs were generated using GraphPad Prism 7.

In a recent analysis of correlation between expression of Wnt ligands and 23 immunosuppressive genes across all cancer types in the TCGA dataset, high levels of Wnt1 has been found to significantly and negatively correlate with CD8 + T cells, showing that it induces immune resistance in lung adenocarcinoma cells and thus immunologically cold tumors (Kerdidani et al., 2019). A meta-analysis based on the data from 1805 NSCLC patients has reported a similar overexpression of Wnt1 and Wnt5a with an inverse correlation to the overall survival of these patients (Jin et al., 2016) (Figure 3).

Another comparative study on NSCLC subtypes has reported that non-canonical Wnt5a was significantly upregulated in the SCC, while ADC was marked by a prominent expression of canonical Wnt7b (Vesel et al., 2017). In SCC, Wnt5a downregulates the ATP-binding cassette (ABC) transporter family members Abcb1 and Abcg2, which are involved in chemotherapy resistance and appear to be upregulated by canonical Wnt signaling when the cells are treated with the chemotherapeutic agent cisplatin (Vesel et al., 2017).

Aberrant pathway activation occurs as a result of mutations or polymorphisms in pathway genes, repression of pathway inhibitors or synergistic effect with other mutations such as Kras (Testa et al., 2018a). For example, Lrp6 rs10845498 polymorphism has been associated with a reduced risk of lung SCC while LRP6 rs6488507 polymorphism synergistically increased the risk of NSCLC in tobacco smokers (Deng et al., 2014). Loss of function mutations in the Wnt-feedback induced cell surface E3 ligases Rnf43 and Znrf3, which bind to Fz and target it for degradation and fusions of the Wnt agonists Rspo2 and Rspo3 have been reported in lung cancer (Katoh and Katoh, 2017). Tobacco smoking, as the main factor responsible for lung cancer, appears to activate Wnt signaling through polycomb-induced repression of the secreted Wnt antagonist Dkk1, resulting in a tumorigenic effect (Hussain et al., 2009). Moreover, concurrent activation of Wnt/β-catenin signaling and expression of the constitutively active Kras mutant *KrasG12D* in the bronchiolar epithelium of the adult mouse lung, significantly increased the tumor number and size (Pacheco-Pinedo et al., 2011).

Misregulation of Wnt signaling in lung cancers have also been associated in many reports with abnormal expression of miRNAs that regulate the membrane components of Wnt pathways. For example, smoking-induced repression of miR-487b -a tumor suppressor miRNA that normally inhibits Wnt5a, Myc, and Kras and upregulates the Wnt antagonists Dkk1, Sfrp1, Sfrp4, and Wif1 to regulate lung stem cells- results in increased proliferation, invasion and metastatic potential of lung cancer cells (Xi et al., 2013). Expression of miR-148a is likewise significantly downregulated in primary cancer tissues of NSCLC patients compared to their adjacent normal lung tissues, and negatively correlates with the expression of Wnt1, a direct target of miRNA-148a (Chen et al., 2017). In contrast, elevated levels of miR-650 in NSCLC has been associated with promotion of cell proliferation and invasion through activation of Wnt1-mediated β-catenin signaling (Tang et al., 2019). Exosomal transfer of miR-1260b has been associated with increased tumor cell invasiveness in lung ADC proliferation through activation of Wnt/β-catenin signaling in neighboring cells (Xia et al., 2020). Interestingly, miR-1260b can induce biosynthesis of ceramides, depletion of which leads to accumulation of Wnt and inhibits Wnt signaling (Pepperl et al., 2013).

#### Targeting Wnt Pathway at the Plasma Membrane for Lung Cancer Therapy

Several therapeutic approaches have been unraveled to suppress lung cancer progression via interfering with Wnt-receptor complex components at RNA or protein levels. For example, aspirin-induced miR-98 expression and long non-coding RNA (lncRNA) MIR503HG have been found to suppress proliferation of lung ADC and NSCLC cells via targeting Wnt1 and thus serve as tumor suppressors (Gan et al., 2019; Lin et al., 2019). Similarly, miR-5587-3p has been reported to suppress Wnt5b, a prognostic biomarker that is highly expressed in lung ADC and positively correlates with metastasis and cancer progression (Zhang et al., 2020). lncRNA AK126698 suppresses Wnt pathway by targeting Fz8 in NSCLC cells, and prevents their proliferation and migration (Fu et al., 2016). miR-135b has been identified to directly target Fz1 at its 3’UTR in NSCLC cells and enhance the chemosensitivity of cisplatin-resistant lung cancer cell lines (Su et al., 2016). On the other hand, antimalarial compounds artemisinin, dihydroartemisinin and artesunate can specifically suppress Wnt pathway by decreasing the protein level of Wnt5a/b (Tong et al., 2016). Qiyusanlong (QYSL) decoction, a formula composed of ten different traditional Chinese medicine, likewise reduces the protein levels of Wnt1, Wnt 2, and Wnt 5a, alone or with Cisplatin (Tong et al., 2018).

### Colorectal Cancer

Colorectal cancer (CRC) ranks second among cancer-related deaths worldwide and majority of CRCs arise sporadically in patients with no family history of disease (Brenner et al., 2014). Hyperactivation of the Wnt pathway due to mutational inactivation of the APC tumor suppressor is thought to be the initiating event and key oncogenic driver in most sporadic and familial CRCs (Schatoff et al., 2017). Furthermore, mutations in both the components and the modulators of Wnt-receptor complex are frequently associated with CRC (Kuipers et al., 2015; Katoh and Katoh, 2017; Testa et al., 2018b). The Cancer Genome Atlas (TCGA) consortium report reveals that Wnt signaling is altered in up to 93% of all sporadic CRCs with at least one and up to sixteen alteration(s) in Wnt pathway components including APC, CTNNB1, TCF7L2, DKK family members, AXIN2 and the pathway negative regulator FAM123B/WTX (Cancer Genome Atlas Network, 2012).

Wnt2 and its receptor Fz7 have been found to be expressed at high levels in CRC as compared to normal colonic mucosa (Holcombe et al., 2002; Kalhor et al., 2018). Wnt3, Wnt6 and Wnt11 are likewise upregulated in CRC in correlation with poor survival rate, and when downregulated proliferation and migration are suppressed and apoptosis is induced (Zheng and Yu, 2018; Gorrono-Etxebarria et al., 2019; Nie et al., 2019; Peng et al., 2019) (Figure 3). Polymorphic adenoma-like protein 2, a zinc finger transcription factor, is also overexpressed in CRC and can promote Wnt6 expression by binding to its promoter region (Li et al., 2019b). The tubulin acetyltransferase αTAT1 promotes CRC progression via regulating subcellular localization of β-catenin and inducing expression of Wnt1 (Oh et al., 2017). Interestingly, Wnt1 is downregulated in response to *Salmonella* infection in CRC and this inhibits cancer cell invasion and migration (Wang et al., 2018). High levels of Wnt5a and its receptor Ror2 have been associated with drug resistance in CRC by concomitant induction of non-canonical Wnt signaling and suppression of canonical Wnt signaling (Bordonaro et al., 2011). Apart from these Wnt ligands, various Fz receptors including Fz4, Fz7, and Fz10, are also upregulated in CRC and their elevated levels have been associated with increased stemness, metastasis and recurrence (Cancer Genome Atlas Network, 2012; Ye et al., 2019; Chi et al., 2020). Wnt co-receptor Lrp6 is likewise significantly upregulated in many tumoral tissues of CRC in correlation with high malignancy and poor prognosis (Rismani et al., 2017). Various Lrp6 polymorphisms such as T867A, N789S, W239L have also been associated with susceptibility to early-onset CRC (de Voer et al., 2016).

Several membrane proteins have been reported to regulate Wnt signaling activity in CRC. For example the low-density lipoprotein receptor-related protein 1B (Lrp1b), which is downregulated in CRC, can suppress the growth and migration of cancer cells via inhibiting the interaction between Dvl2 and the Axin and hence the Wnt/β-catenin signaling (Wang et al., 2017). Expression of the cystic fibrosis transmembrane conductance regulator (Cftr) gene is likewise reduced in CRC, and this reduction enhances Wnt/β-catenin signaling via promoting interaction of Dvl-2 with the plasma membrane (Strubberg et al., 2018). On the contrary, the elevated expression of the type I transmembrane protein CUB-domain containing protein 1 (CDCP1) in CRC has been associated with high metastasis via promoting nuclear localization of β-catenin and Wnt signaling activity (He et al., 2020).

#### The R-Spondin/Lgr5/Rnf43 Module in CRC

Wnt signaling is essential for normal intestinal function due to its roles in maintenance, proliferation and differentiation of intestinal stem cells. In particular, Lgr5 + intestinal stem cells exhibit high levels of canonical Wnt pathway activity reinforced by Rspo1-4 that drives a physical interaction between Lgr4/5 and Rnf43/Znrf3 (de Lau et al., 2014). Rspo fusions with protein tyrosine phosphatase receptor type K (PTPRK-RSPO3) and eukaryotic translation initiation factor subunit E (EIF3E-RSPO2) are frequently observed in colorectal traditional serrated adenomas and characterized by Rspo overexpression and activation of Wnt signaling (Sekine et al., 2017; Hashimoto et al., 2019). In contrast, Rspo2 appears to inhibit CRC metastasis by competing with the tumor-promoting Wnt5a for binding to Fz7 and thus antagonizing Wnt5a-driven non-canonical Wnt signaling (Dong et al., 2017). Although the majority of the studies conducted in primary clinical tissue have suggested a higher expression of the Rspo receptor Lgr5 in CRC cells relative to the adjacent normal tissue, a tumor-promoting role and enhanced chemoresistance, several studies have reported a potential tumor suppressive function of Lgr5 in CRC progression (Hsu et al., 2013; Morgan et al., 2018). *N*-terminal mutations of Rnf43, one of the most commonly mutated genes in CRC, have also been linked with enhanced Wnt/β-catenin signaling activity in colon cancer while *C*-terminal truncation mutants act similarly to the wild-type Rnf43 (Giannakis et al., 2014; Li et al., 2020).

#### Targeting Wnt Pathway at the Plasma Membrane for Colon Cancer Therapy

Due to their potential function as tumor suppressors mentioned above, Lrp1b and Ctfr might offer promising strategies for the treatment of colon cancer. LiCl treatment also inhibits CRC cell proliferation via concomitant induction of the non-canonical ligand Wnt9 and suppression of β-catenin expression (Ali et al., 2016). Combination of inositol hexaphosphate and inositol can also reduce Wnt/β-catenin signaling via downregulating Wnt10b and β-catenin and suppress liver metastasis of CRC (Liu et al., 2020). Different microRNAs have been shown to affect CRC tumorigenesis as well. While miR-140-5p and miR-185 target Wnt1 and act as tumor suppressors, miR-410 targets Dkk1 and thus functions as an oncogene in CRC (Zhang et al., 2018; Wang et al., 2019b; Yeon et al., 2019). These findings reveal that miRNAs can be used as prognostic markers and to produce potential therapeutic agents for CRC patients.

### Liver Cancer

Liver cancer is the fourth common cancer-related death in the world. Hepatocellular carcinoma (HCC), also referred to as hepatoma, is the most common type of liver cancer, constituting approximately 90% of all liver cancers. The molecular events that take place during multi-step initiation and progression of HCC are only partially understood. HCCs are broadly classified into the “proliferation class” and the “non-proliferation class” (Caruso et al., 2019). The proliferation class is further subdivided into the “Wnt-TGFβ subclass” with activated Wnt and TGFβ pathways and the “progenitor subclass” characterized by several features including overexpression of hepatic progenitor markers and mutations in AXIN1 (Rebouissou and Nault, 2020). On the other hand, the “non-proliferation class” of HCC includes tumors that are more heterogeneous, less aggressive, more differentiated with hepatocyte-like features, and contains at least two subclasses (Rebouissou and Nault, 2020). The most well described subclass is characterized by mutations in the β-catenin gene CTNNB1, leading to highly activated Wnt/β-catenin pathway, along with the TERT promoter and TP53 mutations (Yang et al., 2019a; Rebouissou and Nault, 2020). Thus, Wnt/β-catenin signaling is aberrantly activated in approximately 50% of HCC cases, in association with increased proliferation and inflammation, malignant tumor progression, poor prognosis, immune escape, and resistance to therapy (Yang et al., 2013; Khalaf et al., 2018; Jiang et al., 2019b; Ruiz de Galarreta et al., 2019).

Wnt-receptor complex components have been largely identified to take part in hepatocarcinogenesis. For example, several Fz receptors and the canonical Wnt ligands Wnt3 and, to a lesser extent, Wnt10b are strongly upregulated in a variety of HCC cells with different expression levels of hepatocyte lineage, epithelial and mesenchymal markers (Kim et al., 2008; Yuzugullu et al., 2009). Wnt2b, Wnt4, Wnt5a, Wnt5b, Wnt7b, Wnt8b, and Wnt9b are among the other ligands that have significantly increased expression in HCC cell lines (Yuzugullu et al., 2009) (Figure 3). Wnt1 is also highly expressed in HCC cell lines and has been associated with increased tumor recurrence after curative tumor resection in HBV- and HCV-related HCC patients (Lee et al., 2009; Wei et al., 2009). However, Wnt5a and Ror2 have been reported to be downregulated in HCC tissues with a poorer prognosis than HCC patients with elevated Wnt5a and Ror2 expression (Geng et al., 2012). In another study, Wnt5a overexpression was also found to decrease cell proliferation and tumor size in HCC, supporting that Wnt5a may serve as a tumor suppressor in HCC (Wang et al., 2019a). Another non-canonical ligand Wnt11a likewise decreases in HCC and its ectopic expression could suppress cell motility and migration via activation of RhoA/Rho kinase (Toyama et al., 2010). In addition to the Wnt ligands, high levels of Fz2, Fz7, Lrp5, and Lrp6 in HCC have also been found to be promote cell proliferation, migration, invasion, and EMT (Merle et al., 2004; Yuzugullu et al., 2009; Tung et al., 2012; Ou et al., 2019).

#### Targeting Wnt Pathway at the Plasma Membrane for Liver Cancer Therapy

In search of novel potential therapeutic targets for HCC, the tumor-promoting function of Fz7 could be effectively reverted by small interfering RNAs that suppressed proliferation and metastasis of HCC cells and enhanced their apoptosis and sensitivity to chemotherapeutic drugs (Chen et al., 2016, 2018; Xue et al., 2018). Similarly, miR-542-3p, a common tumor-suppresser that is also downregulated in HCC tissues and cell lines, has been shown to inhibit HCC cell growth by targeting Fz7 and may thus represent a novel therapeutic target for HCC (Wu et al., 2017). An anti-Wnt1-antibody has been found to inhibit Wnt/β-catenin signaling and tumor growth in a xenograft mouse model (Wei et al., 2009). In contrast, anti-Wnt1 suppressed proliferation and apoptosis, but did not affect tumor size and growth in diethylnitrosamine-induced hepatocellular adenomas (Sklavos et al., 2018). Interestingly, garlic-derived compound S-allylmercaptocysteine reduced HCC tumorigenesis by directly targeting Lrp6 at the plasma membrane (Xiao et al., 2018).

### Breast Cancer

Breast cancer is the leading cause of cancer death among females worldwide (Bray et al., 2018). Breast cancer is classified into three main subtypes based on the expression of estrogen receptor (ER) or progesterone receptor (PR) and amplification of the human epidermal growth factor 2 (ERBB2, commonly referred to as HER2): hormone receptor positive/ERBB2 negative (HR+/ERBB2-), ERBB2 positive (ERBB2+; HR+ or HR-) and triple-negative (lacking all three molecular markers) (Waks and Winer, 2019). Wnt signaling activity has been reported to increase in both cell lines and patient-derived metastatic cells of breast cancer and positively correlate with the ER expression (Lamb et al., 2013). Wnt/β-catenin pathway activation, characterized by reduction of membranous β-catenin with its concomitant nuclear accumulation, appears to correlate with poor clinical outcome in triple-negative breast cancer (TNBC) and basal-like breast cancer (BLBC), an aggressive subtype of breast cancer characterized by strong expression of basal markers such as cytokeratins (Khramtsov et al., 2010; Geyer et al., 2011; Xu et al., 2015).

The role of plasma membrane in breast cancer has been unraveled in a study that characterized the role of cell adhesion protein CD44, which appears to localize preferably to the lipid rafts due to palmitoylation. Raft affiliation of CD44 was observed to be higher in non-invasive breast cell lines, while decreasing in highly invasive cell lines or in case of mutagenesis of palmitoylation sites, suggesting that lipid raft association is a key regulatory mechanism in cancer cell migration (Babina et al., 2014). Interestingly γ-Tocotrienol, a natural isoform of vitamin E, appears to disrupt the lipid raft integrity, suppress Wnt/β-catenin signaling pathway and reduce cell motility in breast cancer (Ahmed et al., 2016). Thus, it will be very interesting to test how lipidation of Wnt and its receptors or raft association of Wnt-receptor complex influence migration and metastasis of these cells.

#### Misregulation of Wnt-Receptor Complex Components in Breast Cancer

In a network correlation analysis of expression of >100 Wnt pathway components in healthy and cancerous breast tissues, the strong coherence in expression levels of the Wnt ligands and Fz receptors observed in the healthy breast tissue is dramatically lost in TNBC tissue and also varies widely in TNBC and non-TNBC (Koval and Katanaev, 2018) (Figure 3). Wnt4 and Wnt16 have been reported to be significantly upregulated in TNBC recurrence (Tsai et al., 2015). Wnt3a, 5a, 5b, 9a, and 11 are preferentially overexpressed due to gene amplifications in BLBC (Shi et al., 2014; Jiang et al., 2019a). Among them, Wnt5b has been identified as a key regulatory factor that governs the BLBC phenotype by activating both canonical and non-canonical Wnt signaling (Jiang et al., 2019a). Interestingly, Wnt5a expression was shown to decrease at both mRNA and protein levels in TNBC in association with poor prognosis and Wnt5a signaling was able to suppress tumor growth and metastasis (Borcherding et al., 2015; Zhong et al., 2016). Wnt3a and Wnt7a are likewise upregulated in metastatic breast cancer cell lines in association with poor prognosis and inhibition of Wnt/β-catenin signaling via Wnt1 knockdown could efficiently suppress cell proliferation and tumor growth (Jang et al., 2015; Avgustinova et al., 2016). The receptors Fz2, Fz3, Fz6, Fz7, and Fz10 as well as the co-receptor Lrp6 have also been reported to significantly increase in breast cancer and contribute to mesenchymal-like stemness, invasion, metastasis, and drug resistance (Liu et al., 2010; Yang et al., 2011; Gong et al., 2014; Simmons et al., 2014; Bell et al., 2017; Corda et al., 2017; Yin et al., 2020). Loss-of-function mutations of the tumor-suppressor-like molecules Rnf43 and Znrf3 and elevated expression levels of the Wnt agonists Rspo2 and Rspo4 can also be counted among the prognostic biomarkers of breast cancer (Ciriello et al., 2015; Coussy et al., 2017; Katoh and Katoh, 2017).

#### Targeting Wnt Pathway at the Plasma Membrane for Breast Cancer Therapy

A number of studies have assessed the therapeutic potential of targeting Wnt signaling at the plasma membrane in TNBC. Being an important biomarker of TNBC, Fz7 is one of the well-investigated therapeutic options for breast cancer. A recombinant soluble peptide fragment (rhFzd7) has been shown to antagonize Fzd7 by competitively binding with Wnt3a and exhibit anti-tumor and anti-angiogenesis activities in TNBC (Xie et al., 2018). 2-cyano-3, 12-dioxooleana-1, 9 (11)-dien-28-oic acid-methyl ester (CDDO-Me) likewise targets Fz7 and Lrp6 and significantly inhibited tumor growth in breast cancer (Zhou et al., 2020). miR-142-3p, which is significantly down-regulated in breast cancer tissues, can also suppress Fz7 and thus serve as a tumor suppressor in breast cancer (Jia et al., 2018). A recent *in silico* study have identified several candidate molecules similar to palmitoleic acid that could potentially bind to the Fz7 transmembrane protein and inactivate the Wnt signaling pathway in TNBC cells (Alves Pinto and Freitas Da Silveira, 2020). In addition to Fz7, targeting Wnt ligands, Dkk1 or Lrp6 at the membrane may offer promising treatment options against breast cancer. For example, the antihelminthic niclosamide could sensitize TNBC cells to ionizing radiation (IR) by suppressing Wnt3a/β-catenin mediated radioresistance (Yin et al., 2016). A monoclonal anti-Wnt-1 antibody or Wnt-1 siRNA inhibit could induce apoptosis in a variety of human cancer cell lines including breast cancer (He et al., 2004). The polycomb protein chromobox homolog 7a (CBX7) likewise appears to inhibit breast tumorigenicity by enhancing the expression of the Wnt antagonist Dkk1 (Kim et al., 2015). The polyether ionophore antibiotic salinomycin, the milk thistle flavonolignan silibinin, the natural phenol echinacoside and the natural plant polyphenol rottlerin have all been shown to inhibit Wnt/β-catenin signaling by suppressing the Wnt co-receptor Lrp6 expression and exert anti-tumor effects in TNBC (Lu et al., 2012, 2014; Lu and Li, 2014; Tang et al., 2020).

### Other Cancers

It is noteworthy to mention that aberrant activation of Wnt signaling pathways is obviously not limited to the four cancer types mentioned below. Various Wnt ligands, the agonist Norrin, Fz receptors and the co-receptors Lrp6 and Ror1/2 have been reported to be abnormally expressed and associated with metastatic behavior, cancer progression and chemoresistance in ovarian cancer, glioblastoma multiforme, chronic lymphocytic leukemia, melanoma, multiple myeloma, post-transplant smooth muscle tumor, prostate cancer, pancreatic cancer gastric cancer, oral squamous cell carcinoma, Ewing sarcoma, osteosarcoma, and malignant peripheral nerve sheath tumors (Derksen et al., 2004; Larue and Delmas, 2006; Dissanayake et al., 2007; Yan et al., 2016; Yu et al., 2016; Li et al., 2017, 2019a; Liu et al., 2017; Pridgeon et al., 2017; Sandsmark et al., 2017; Jiang et al., 2018; Sinnberg et al., 2018; Teiken et al., 2018; Yang et al., 2019b; Chehover et al., 2020; El-Sehemy et al., 2020; Frenquelli et al., 2020; Kotrbova et al., 2020). Thus, a thorough understanding of misregulation of Wnt signaling pathways at the plasma membrane will pave the way for new therapeutic approaches for cancer.

### Inhibition of Wnt Pathway for Cancer Therapy

**FIGURE 4:**
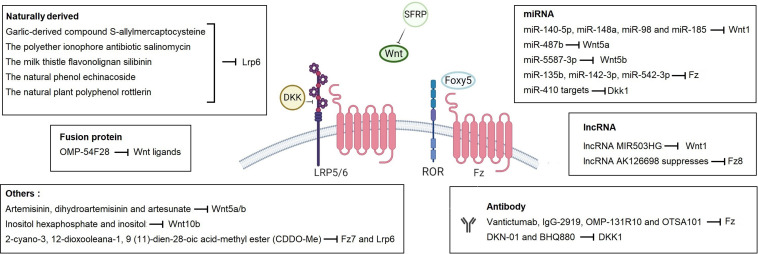
Therapeutic molecules that target Wnt signaling pathway at the level of the plasma membrane. Different types of molecules are given with their target pathway component in boxes. Created with BioRender.com.

Biological inhibitors and small molecules related with the Wnt pathways are widely exploited in therapeutic approaches to human diseases, including cancer, that have increased Wnt signaling activity. With its pivotal role in initiation and tight control of Wnt signaling activity, components and modulators of the Wnt-receptor complex constitute promising drug targets (Katoh and Katoh, 2017; Taciak et al., 2018; Goldsberry et al., 2019) (Figure 4). For example, Ipafricept (OMP-54F28), a recombinant fusion protein comprised of the Fz8 cysteine-rich domain and human IgG1 Fc fragment, acts as decoy receptor for Wnt ligands and exhibits antitumor activity (Le et al., 2015; Jimeno et al., 2017). OTSA 101-DTPA90Y, Vantictumab (OMP-18R5) and IgG-2919 are monoclonal antibodies targeting different Fz receptors and decrease tumor growth in different cancers (Gurney et al., 2012; Nielsen et al., 2015; Steinhart et al., 2017). The monoclonal antibodies DKN-01 and BHQ880 target DKK1 and likewise exert anti-tumorigenic activity in relapsed or refractory cancers including NSCLC, multiple myeloma and gastrointestinal cancers (Fulciniti et al., 2009; Edenfield et al., 2014; Iyer et al., 2014; Bendell et al., 2016). Rosmantuzumab (OMP-131R10) targets Rspo3 and evokes favorable responses against solid tumors and CRC (Diamond et al., 2016). The small molecule inhibitor KAN0439834, antibodies Cirmtuzumab (UC-961), ROR1-CD3-DART, APVO425, and ROR1R-CAR-T cells target Ror-1 with promising effects on different types of cancers (Berger et al., 2015; Yu et al., 2016; Katoh and Katoh, 2017). LGK974, ETC-159 (ETC1922159), RXC004, CGX1321, GNF-6231, XNM7201, IWP-2, WNT974, and WNT-C59 are all Porcupine inhibitors that are in the preclinical or phase I/II stage (Katoh and Katoh, 2017; Taciak et al., 2018; Goldsberry et al., 2019). When combined with pan-PI3K inhibitor GDC-0941, porcupine inhibitor ETC-159 has been shown to potently suppress *in vivo* tumor growth in pancreatic cancer (Zhong et al., 2019). Finally, Foxy-5, a small peptide that mimics Wnt5a, is considered to disrupt the migration and invasion of epithelial cancer cells and exhibit anti-metastatic impact in metastatic breast, colorectal and prostate cancers (Canesin et al., 2017).

Clinical trials with several of these drugs have reported various adverse effects. For example, while being well tolerated by the patients with solid tumors, Ipafricept caused at least one of the treatment-emergent adverse events including dysgeusia, decreased appetite, fatigue, muscle spasms, and nausea, each of which were observed in at least 20% of patients (Jimeno et al., 2017). Different dose combinations of Vantictumab have been tested for 23 patients with advanced solid tumors, and likewise caused fatigue, nausea, vomiting, abdominal pain, constipation, and diarrhea as most common related adverse effects (Smith et al., 2013). First-in-human study of OTSA-101 on metastatic synovial sarcomna patients have reported lymphopenia, anemia, leucopenia, asthenia, hemoptysis, thrombocytopenia, neutropenia, and anorexia in some or all patients depending on the applied doses (Giraudet et al., 2018). The anti-DKK1 antibody DKN-01 also appears to cause adverse effects including cough, peripheral neuropathy, alopecia, leukopenia, neutropenia and fatigue in patients of refractory esophageal cancer or gastro-esophageal junction tumors (Bendell et al., 2016). Thus, further detailed investigations are absolutely necessary to assess the potential of Wnt inhibitors in therapeutic interventions.

## Conclusion

The plasma membrane composition and organization play an important role in regulation of Wnt signaling by controlling ligand-receptor interaction and signal initiation. Since plasma membrane is highly dysregulated in cancer, it is essential to consider the unique organization of the Wnt-receptor complex for specific and effective targeting of the cancer cell. Understanding complex molecular interactions underlying Wnt-mediated cellular events at the plasma membrane has the potential to reveal attractive drug targets in cancers, and potentially other diseases, where Wnt signaling is extensively involved.

## Author Contributions

GO, YA, and MK wrote the manuscript and prepared the figures. EE contributed to the discussion. All authors contributed to the article and approved the submitted version.

## Conflict of Interest

The authors declare that the research was conducted in the absence of any commercial or financial relationships that could be construed as a potential conflict of interest.
